# A systems serology approach to the investigation of infection-induced antibody responses and protection in trachoma

**DOI:** 10.3389/fimmu.2023.1178741

**Published:** 2023-05-23

**Authors:** Amber Barton, Ida Rosenkrands, Harry Pickering, Nkoyo Faal, Anna Harte, Hassan Joof, Pateh Makalo, Manon Ragonnet, Anja Weinreich Olsen, Robin L. Bailey, David C. W. Mabey, Frank Follmann, Jes Dietrich, Martin J. Holland

**Affiliations:** ^1^ Department of Clinical Research, London School of Hygiene and Tropical Medicine, London, United Kingdom; ^2^ Department of Infectious Disease Immunology, Statens Serum Institut, Copenhagen, Denmark; ^3^ Medical Research Council Unit The Gambia at London School of Hygiene and Tropical Medicine, Banjul, Gambia

**Keywords:** trachoma, *Chlamydia trachomatis*, antibody, systems serology, IgG, humoral immunity

## Abstract

**Background:**

Ocular infections with *Chlamydia trachomatis* serovars A–C cause the neglected tropical disease trachoma. As infection does not confer complete immunity, repeated infections are common, leading to long-term sequelae such as scarring and blindness. Here, we apply a systems serology approach to investigate whether systemic antibody features are associated with susceptibility to infection.

**Methods:**

Sera from children in five trachoma endemic villages in the Gambia were assayed for 23 antibody features: IgG responses towards two *C. trachomatis* antigens and three serovars [elementary bodies and major outer membrane protein (MOMP), serovars A–C], IgG responses towards five MOMP peptides (serovars A–C), neutralization, and antibody-dependent phagocytosis. Participants were considered resistant if they subsequently developed infection only when over 70% of other children in the same compound were infected.

**Results:**

The antibody features assayed were not associated with resistance to infection (false discovery rate < 0.05). Anti-MOMP SvA IgG and neutralization titer were higher in susceptible individuals (*p* < 0.05 before multiple testing adjustment). Classification using partial least squares performed only slightly better than chance in distinguishing between susceptible and resistant participants based on systemic antibody profile (specificity 71%, sensitivity 36%).

**Conclusions:**

Systemic infection-induced IgG and functional antibody responses do not appear to be protective against subsequent infection. Ocular responses, IgA, avidity, or cell-mediated responses may play a greater role in protective immunity than systemic IgG.

## 1 Introduction

The obligate intracellular bacterium *Chlamydia trachomatis* is a major human pathogen with a unique biphasic life cycle, alternating between intracellular reticulate bodies and extracellular elementary bodies (EBs). During the EB phase of its life cycle, 61% of the *C. trachomatis* outer membrane consists of major outer membrane protein (MOMP) by mass (1), a transmembrane porin and target for surface-binding antibodies. MOMP consists of five constant and four variable domains, of which the surface-exposed variable domains define the *C. trachomatis* serovar (2). Whereas serovars (Sv) A–C cause the childhood ocular disease trachoma, D-K cause sexually transmitted urogenital disease and L1–L3 lymphogranuloma venereum (3). In the 1960s, several whole-organism vaccines were found to confer short-term serovar-specific immunity to trachoma (4), and MOMP-based subunit vaccines hold great promise as another tool in moving past elimination towards eradication. However, no vaccines against *C. trachomatis* have yet been licensed.

While cell-mediated Th1 responses are thought to be important for protection against trachoma (5), the role of humoral responses in protection is not well established. In human challenge studies, primary infection with ocular *C. trachomatis* conferred only incomplete, serovar-specific immunity (6). Repeated infections are therefore common, resulting in conjunctival inflammation in the short term (“active trachoma”), and scarring and trichiasis in the long term (5).

The relationship between trachoma and humoral immunity in field studies is complex. Firstly, systemic antibody responses detected in the serum may have a different effect to local responses detected in the tears. For example, in one study, serum anti-EB IgG was higher in controls than in those with active trachoma (7), suggesting a protective association, while in another, tear anti-EB IgG was a risk factor for active trachoma (8). Antibody isotype also plays a role: anti-EB IgG is higher in those with trachomatous scarring, while anti-EB IgA is lower (9). No significant differences in MOMP-specific serum IgA or IgG have been observed between children with or without active trachoma, or between adults with or without trachomatous scarring (7). Likewise, no significant differences in MOMP-specific tear IgA were found between clinical groups in Nepal (10).

Given the previous conflicting relationships between humoral responses and trachoma, we hypothesized that antibody functional characteristics or antigen specificity may be important for protection against infection. We therefore used a data-driven systems serology approach (11) with the aim of finding individual antibody features or overall antibody profiles associated with resistance to ocular *C. trachomatis*. This strategy has previously been successful in identifying multivariate signatures and new features of protective responses to typhoid vaccination (12), and distinguishing those with controlled latent tuberculosis from those with active tuberculosis (13). Here, we apply the systems serology approach to a longitudinal study of ocular *C. trachomatis* infection. We find that IgG responses to antigens from different serovars correlate strongly with one another, despite serovar B predominating in the local area. We then investigate the association between individual antibody features and susceptibility, and assess whether susceptible and resistant participants have distinct serum antibody profiles.

## 2 Materials and methods

### 2.1 Ethical approval

The study design and procedures were approved by the joint Gambian Government-Medical Research Council Ethics Committee and the Ethics Committee of the London School of Hygiene & Tropical Medicine (MRC SCC: 745/781; MRC SCC L2008.75; LSHTM: 535) and was conducted in accordance with the Declaration of Helsinki. Verbal consent was obtained from community leaders, and written informed consent was obtained from all study participants’ guardians on their behalf. A signature or thumbprint was considered an appropriate record of consent in this setting by the above ethical bodies. Archival sample storage and secondary use in related immunological studies were included as part of the consent and approvals.

### 2.2 Study recruitment and sample collection

Local information provided by the Gambian National Eye Care Program was used to identify which villages to target for initial screening. Nine villages were selected after a trachoma rapid assessment survey, carried out on the Western and North Bank Regions of the Gambia before the study, found active trachoma in >20% of school age-children. A total of 345 children aged 4–15 years from 31 family compounds were visited at baseline and fortnightly for 28 weeks (14). The village health worker in each settlement contacted households in the cohort the day before each sample collection time point, and households were visited the same day of the week every 2 weeks. These village health workers (“nyateros”, or friends of the eye) were trained in primary eye care and health promotion as part of the Gambian national eye care program. At the time of sample collection, all participants were observed by the community ophthalmic nurse, field assistants, and health visitors as otherwise healthy. Minor unrelated conditions were treated by the nurse. Samples were not collected from those requiring referral to regional health posts for any other treatment. The study took place prior to introduction of mass drug administration, but children with intense inflammatory trachoma were treated upon diagnosis, and at the end of the study, each member of the household in which participants were resident was offered treatment with oral azithromycin. Where possible, if study participants were absent from the village, attempts were made to revisit the village the following day, with approximately 70% completeness of observations relative to those initially planned. At each visit, conjunctival swabs were collected in RNAlater (Ambion Europe Ltd, Huntingdon, UK), and stored at −20°C (15). *C. trachomatis* infection and load were determined by 16S ribosomal RNA quantitative polymerase chain reaction (PCR) as described previously (16). DNA for OmpA serovar determination was extracted from swabs by one of two methods: either by the QIAamp DNA Mini Kit, as previously described (17), or by the Roche CT/NG amplicor kit followed by concentration of positive samples using a QIAamp DNA Mini Kit (18).

A total of 160 venous blood samples (10 ml) were collected at baseline in BD Vacutainer Lithium Heparin Tubes (19). Samples were transported to MRC laboratories within 4 h, in a sealed box at ambient temperature. Plasma was separated by density gradient centrifugation using Lymphoprep (Axis-Shield Ltd, Kimbolton, UK) and stored at −30°C. Serological assays were carried out on a subset of 93 samples.

### 2.3 OmpA serovar

OmpA serovar was determined by ompA Sanger sequencing or *C. trachomatis* whole gene sequencing. Prior to Sanger sequencing, conjunctival swab DNA samples underwent either one round of PCR or two rounds of PCR *via* a nested PCR approach, using a Veriti Thermocycler (Thermo Fisher) and following the procedure outlined in Andreasen et al. (18). PCR products were sequenced at Macrogen or Source Bioscience and were compared against all available *C. trachomatis* sequences available in NCBI using BLAST to determine serovar.

Whole genome sequencing was performed as previously described (20) and utilized the SureSelectXT Low Input kit. Processing and analysis of sequenced reads were performed as previously described (21). Raw reads were trimmed and filtered using Trimmomatic. Filtered reads were aligned to a reference genome (A/Har13) with Bowtie2, and variants were called with SAMtools/BCFtools. Multiple genome and plasmid alignments were generated using progressive Mauve, and multiple gene alignments were generated using MUSCLE. Complete sequences of ompA were obtained using a reference-based assembly method also previously described (21). Serovar was assigned using maximum blastn homology against all published *C. trachomatis* sequences.

### 2.4 *C. trachomatis* culture

EB antigens were produced in-house through propagation in either HEp-2 or HeLa-229 cells at the London School of Hygiene and Tropical Medicine and Statens Serum Institut, respectively. HeLa cells originated from a glandular adenocarcinoma of the cervix (22), while HEp-2 cells, originally reported as epidermoid carcinoma, are likely derived from HeLa contamination (23, 24).

For EB IgG quantification by area under the dilution curve, EBs from *C. trachomatis* SvA (clinical strain A/2497), SvB (Tunis-864), and SvC (C/TW-3) were prepared by infecting HEp-2 cells at an MOI of 0.5–1 in the presence of MEM medium with 10% with fetal calf serum (FCS). Cells were centrifuged at 1,800 rpm for 1 h at 37°C, then incubated at 37°C and 5% CO_2_ for 2 h. The medium was then replaced by MEM with 10% FCS, 1 µg/ml cycloheximide, 0.5% glucose, and 10 µg/ml gentamycin. EBs were purified by washing cells with 5 ml of HBSS, incubating with 0.05% trypsin/0.02% EDTA in PBS, then resuspending cells in culture medium. Resuspended cells were centrifuged at 3,750 rpm for 10 min and then resuspended in sucrose-phosphate medium (68.5 g/L sucrose, 2.07 g/L dipotassium hydrogen phosphate, 1.1 g/L potassium hydrogen phosphate, 5% FCS, 0.5% phenol red, 0.05 g/L streptomycin sulfate, 0.1 g/L vancomycin hydrochloride, and 625 µg/L amphotericin B) on ice. Cells were sonicated and then EBs were purified by ultracentrifugation on a 20% urografin gradient.

For neutralization and phagocytosis assays, EBs from *C. trachomatis* SvB (Tunis-864) were prepared by infecting HeLa-229 cells (ATCC CCL-2.1, RRID : CVCL_1276) in the presence of RPMI 1640 medium (Gibco, Australia) containing 5% heat-inactivated FCS and 50 μg/ml gentamycin (Gibco, Australia) as previously described (25). Briefly, after harvesting the bacteria by two high-speed centrifugation steps, the suspended bacteria were sonicated, further purified on renografin cushion by ultracentrifugation, resuspended in 250 mmol/L sucrose, 10 mmol/L NaH_2_PO_4_, and 5 mmol/L l-glutamic acid (SPG buffer), and stored at −80°C. The inclusion-forming units (IFUs) of the batches were quantified by titration in HeLa-229 cells.

### 2.5 EB and MOMP IgG quantification by area under the dilution curve

Recombinant MOMP SvA, SvB, and SvC were produced based on the amino acid sequences (NCBI CAX09353.1, ABB51015.1, and WP_024067253.1) with an added N-terminal six-histidine tag. Synthetic DNA constructs were codon optimized for expression in *Escherichia coli*, followed by insertion into the pJexpress 411 vector (Atum). Purification was done essentially as described elsewhere (26). Briefly, expression was induced by IPTG in *Escherichia coli* BL-21 (DE3) cells transformed with the synthetic DNA constructs. Inclusion bodies were isolated and extracts were loaded on a HisTrap column (GE Healthcare), followed by anion exchange chromatography on a HiTrap Q HP column and dialysis to 20 mM tris-HCl, pH 8.0. Protein concentrations were determined by the bicinchoninic acid protein assay.

For EB-specific IgG assays, test and positive control serum were assayed at four different dilutions (1:10, 1:100, 1:1,000, and 1:10,000, each in singlicate). For MOMP-specific IgG assays, test and positive control serum were assayed at four different dilutions (1:10, 1:50, 1:500, and 1:10,000, each in singlicate). MOMP-specific IgG was also validated independently at a different site (see *Section 2.6*). Diluted serum (45 µl) was added to each well and incubated at room temperature for 2 h. Plates were then washed four times. Anti-human IgG HRP antibody (Anti-Human IgG-Peroxidase from Sigma) was diluted 1:30,000 in blocking buffer, and 100 µl was added to each well. Following incubation at room temperature for 1 h, plates were washed four times. 1-Step Ultra TMB-ELISA Substrate Solution (100 µl) was added per well and the plate developed for 30 min in the dark. One hundred microliters of 1 M sulfuric acid was added per well to stop the reaction. Plates were read at 450 nm and 630 nm. The absorbance at 630 nm was subtracted from the absorbance at 450 nm to give a background-adjusted absorbance. For each plate, absorbances were normalized relative to a positive control, fit to a 4-parameter logistic curve using the drc package in R, and the area under the curve was calculated to give a measure of antigen-specific IgG quantity.

### 2.6 MOMP peptide-specific IgG and validation of MOMP IgG response

Synthetic peptides (20–22mers) representing variable domain (VD) VD1, VD2, preVD3, VD3, and VD4 from SvA, B, and C were produced by Genecust (**Table S1**). Maxisorp Plates (Nunc, Denmark) were coated overnight with either recombinant MOMP (1 µg/ml) or synthetic peptides (10 µg/ml) in carbonate buffer. Plates were washed with PBS containing 0.2% Tween-20 and blocked with 2% skimmed milk in PBS. Serum samples were diluted 1:50 in PBS with 1% skimmed milk, and as a secondary antibody, we used anti-human IgG HRP antibody (Agilent P0214) diluted 1:8,000 in PBS + 1% skimmed milk. TMB Plus (Kem-En-TEC) was used for detection and plates were read at 450 nm with subtraction of the absorbance value measured at 620 nm. The MOMP peptide analysis was performed for 37 samples with a positive response to either recombinant SvA, SvB, or SvC MOMP.

### 2.7 *C. trachomatis* neutralization

For 91 out of 93 samples, the neutralization assay was performed essentially as described (27, 28). Briefly, Syrian golden hamster kidney (HaK) cells (ATCC CCL-15), established in 1993 as the standard for *C. trachomatis* neutralization assays (27), were seeded in 96-well flat-bottom microtiter plates. Serum samples were heat-inactivated at 56°C for 30 min and diluted in SPG buffer to create twofold dilution series starting from 20-fold dilution. The diluted *C. trachomatis* SvB stock was mixed 1:1 with the diluted plasma samples and incubated for 45 min at 37°C, 5% CO_2_. The mixture was used for infection of HaK cells for 2 h at 35°C, and thereafter, the cells were incubated for 24 h. Chlamydial inclusions were visualized by staining with polyclonal rabbit anti-recombinant CT043 serum, followed by Alexa Fluor 488–conjugated goat anti-rabbit immunoglobulin (Life Technologies). Cells were stained with propidium iodide (Invitrogen) and IFUs were counted by an ImageXpress Pico automated cell imaging system (Molecular Devices, San Jose, CA) as previously described (28). A positive and negative reference pool from rabbits immunized with Hirep1 (25) were included on all plates. Percent specific neutralization was calculated as [(No sample control IFU − sample IFU)/No sample control IFU] × 100 for each dilution, and the serum dilution giving a 50% reduction in IFU was named reciprocal 50% neutralization titer (NT_50_). Reciprocal NT_50_ values were calculated based on a five-parameter logistic curve using the package “drc” in R. For samples where no titer could be calculated, the samples were assigned an NT_50_ titer of 10 (half the value of the lowest dilution).

### 2.8 Fc-receptor-dependent phagocytosis

The phagocytosis assay has been previously described (29), with the modification that *C. trachomatis* was not prelabeled with carboxyfluorescein diacetate succinimidyl ester in this study. Briefly, PLB-985 cells, a human myeloid leukemia line capable of differentiation to a phagocytic neutrophil-like phenotype, were cultured in RPMI 1640 with 1% L-glutamine, 1% HEPES, 1% pyruvate, 1% Non-Essential Amino Acids Solution, 10 µg/ml gentamicin, and 10% heat-inactivated FBS at 37°C and 5% CO_2_. PLB-985 cells were stimulated with 100 mM N,N-dimethylformamide (DMF; Sigma-Aldrich) for 5 days to induce differentiation into a neutrophil-like phenotype. Serum samples were diluted 1:100 and *C. trachomatis* SvB bacteria were diluted in the PLB-985 culture medium, mixed in a 1:1 ratio, and then incubated for 40 min at 37°C on a rocker table. Forty microliters of the serum/bacteria suspension was then mixed with 100µl of the DMF stimulated PLB-985 cells (2 × 10^6^ cells/ml) at an MOI of 2.8. Following incubation for 4 h at 37°C on a rocker table, cells were washed with PBS and then stained with eBioscience Fixable Viability Dye eFluor 520. After 15 min, cells were washed with FACS buffer (PBS with 2% FBS, 0.1% sodium azide, and 1 mM EDTA) and fixed using BD Cytofix. Thereafter, cells were incubated with monoclonal mouse anti-*C. trachomatis* LPS (Abnova) in PBS followed by incubation with goat-anti-mouse IgG conjugated with AlexaFluor 647. Samples were measured using a BD FACSCanto. Live singlet PLB-985 cells were gated and the *C. trachomatis* SvB signal was measured in the APC channel. The analysis was performed for a representative subset of 26 samples.

### 2.9 Serology data analysis

Statistical analysis was carried out in the R statistical software environment. Correlation between antibody features was assessed by calculating Spearman’s correlation coefficient for complete observations. Distance was calculated using the as.dist function in R [distance = as.dist(1 − Spearman’s rank correlation coefficient)]. Unsupervised hierarchical cluster analysis was then performed on the distance matrix using the hclust function in R. A test of the correlation coefficient being zero was carried out using the cor.test function.

To find whether any individual antibody features were associated with susceptibility, each antibody feature was centered and scaled, and a logistic regression model with age, sex, and ethnicity as covariates was used. To carry out multivariate analyses, missing data were imputed using the k-nearest neighbors (kNN) method in the preprocessing function of the caret package (30). Briefly, kNN imputation identifies the samples most similar to the sample with missing data (the nearest neighbors), and averages the value from these samples. Data were once again centered and scaled. Principal component analysis was carried out using the prcomp function in R. A partial least squares classification model was built using the caret package, using receiver operating characteristic (ROC) as a metric to choose the optimal model. In this case, a model using two components (linear combinations of the original features) was optimal. The specificity and sensitivity of the model in classifying susceptible individuals were tested using 10-fold cross-validation.

### 2.10 Data availability

The original contributions presented in the study are included in the **Supplementary Material** (**Supplementary Document 1**).

## 3 Results

### 3.1 Infection susceptibility in the study population

**Figure 1:**
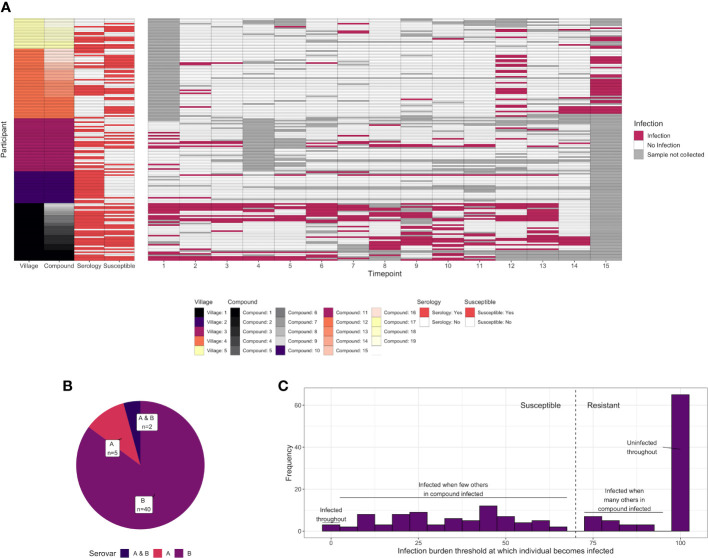
**(A)** *C. trachomatis* infection over 15 time points (2-week intervals) in 160 children from five villages and 19 compounds in the Gambia. Infected time points are highlighted in red, whereas time points where a sample was not collected for a particular participant is shown in gray. The characteristics of each participant are annotated on the left, including village, compound, whether they were deemed susceptible (red = yes), and whether serological assays were carried out (red = yes). Assays were carried out on samples collected at time point T1. **(B)** For 51 individuals for which *C. trachomatis* serovar was successfully identified, the proportion that were infected with serovar A, infected with serovar B, or infected with both at different time points. **(C)** Distribution of the infection burden (% infected) a child’s compound would need to reach before they themselves became infected. A total of 160 individuals from 19 compounds, of which a subset was sampled for systems serology (93/160), are included.

Serological assays were carried out on serum samples from *n* = 93 children living in 19 family compounds within five villages in the Gambia (**Figure S1**). These children were a subgroup of those recruited to a larger longitudinal study [*n* = 345, (14)]. Family compounds in the longitudinal study were visited every 2 weeks for 28 weeks (15 time points) to test for ocular *C. trachomatis* infection. In addition to the 93 children who underwent serological profiling at baseline, a further 67 children in these 19 compounds were followed up longitudinally for infection (**Figure 1A**, total of 160 children in these compounds). By a combination of whole genome sequencing and ompA amplicon sequencing, ompA serovar was successfully determined for at least one time point in 47 individuals, of which 40 were infected with serovar B; 5 with serovar A; and 2 individuals with A and B at separate time points (**Figure 1B**).

Previously, members of this cohort were categorized as susceptible or resistant based on subsequent duration of infection (31). However, the proportion of sampled children who were *C. trachomatis* positive varied over time and between compounds, suggesting that some participants will have been labeled “resistant” through lack of exposure rather than immunity. To address this, for each of the 160 participants in the 19 compounds from which samples were collected, we examined how the percentage of *C. trachomatis* positive children in their compound varied over time. A logistic regression model was used to predict what threshold the infection burden in their compound needed to reach before each individual became infected (**Figure S2**). This threshold followed a bimodal distribution (**Figure 1C**). We therefore considered children who became infected when less than 70% of the other children in their compound were infected to be “susceptible”, and others to be “resistant”.

Of the 93 children that underwent serological profiling at baseline, *n* = 45 were susceptible and *n* = 48 resistant. While the children in these two groups were similar in age, female children were more likely to be susceptible (susceptible group 60% female versus resistant group 31% female, *p* = 0.0069, Fisher test; **Tables 1**, **S2**).

**Table 1:** Characteristics of “susceptible” and “resistant” participants for the 93 samples on which serological assays were performed.

	Susceptible	Resistant
*N*	45	48
Age	7 (IQR 5–10)	8 (IQR 6–12)
% Female	60	31.25
Ethnicity	Mandinka 31%, Manjago 51%, Serahule 7%, Serer 2%, Wolof 9%	Mandinka 56%, Manjago 15%, Serahule 29%

### 3.2 Antigen-specific antibody responses to different serovars correlate closely with one another

**Figure 2:**
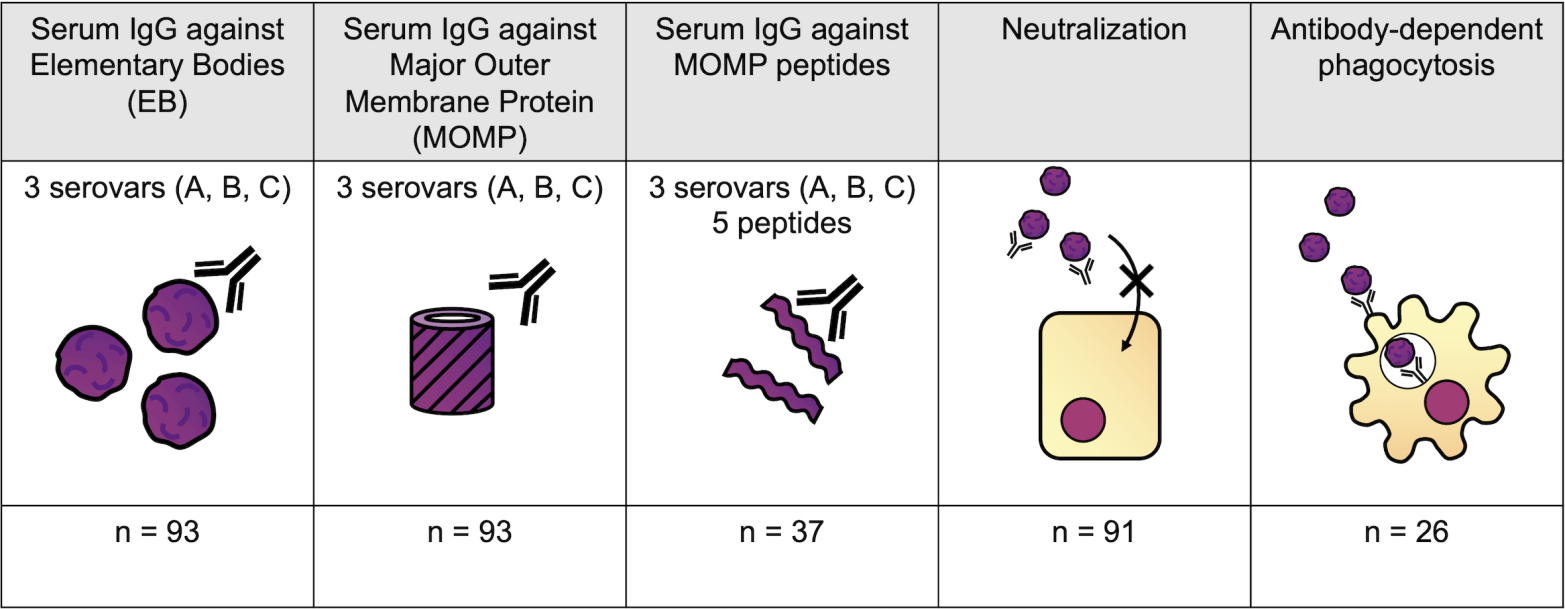
Serological assays performed. Antigen-specific assays measured total IgG specific to *C. trachomatis* EBs, recombinant MOMP, and five peptides within MOMP (serovars A-C). Functional assays measured the ability of serum to neutralize infection of HaK cells, or to induce Fc-receptor-dependent *C. trachomatis* phagocytosis by myeloid PLB-985 cells.

The serological assays carried out are summarized in **Figure 2**. Antigen-specific assays measured total IgG specific to *C. trachomatis* EBs and recombinant MOMP (both calculated by area under the dilution curve), and five peptides within or adjacent to the variable domains (VD) of MOMP (serovars A, B, and C) representing the linear epitopes in these regions. Functional assays measured the ability of serum to neutralize infection of HaK cells with serovar B *C. trachomatis*, or to induce Fc-receptor dependent phagocytosis of serovar B *C. trachomatis* by myeloid PLB-985 cells.

**Figure 3:**
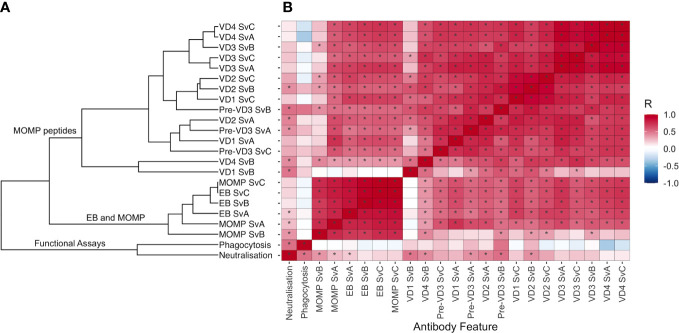
**(A)** Hierarchal clustering of antibody features. Spearman’s rank correlation coefficient was calculated for each pair of features, and distance was calculated using the as.dist function in R [distance = as.dist(1 − Spearman’s rank correlation coefficient)]. Unsupervised hierarchical cluster analysis was then performed on the distance matrix. **(B)** Correlation between different antibody features, colored by Spearman’s rank correlation coefficient. Features are ordered as in **(A)**. Significant correlations (*p* < 0.05) are highlighted by an asterisk.

To elucidate the relationship between different antibody features, Spearman’s rank correlation coefficient was calculated for each pair of features (**Supplementary document 2**). This correlation matrix was used to calculate a distance matrix and hierarchal clustering was carried out. Antibody features were clustered by whether the assay measured functional responses, IgG responses to EB and MOMP, or IgG responses to MOMP peptides (**Figure 3A**). Antigen-specific IgG responses to whole EBs and MOMP closely correlated with one another, regardless of the antigen serovar (**Figure 3B**; Spearman’s rank correlation coefficients ranging from 0.692 to 0.998; median, 0.820). IgG responses to peptides within MOMP were also closely correlated (Spearman’s rank correlation coefficients ranging from 0.160 to 0.979; median, 0.715), although the response to VD1 SvB was a notable outlier, with generally low responses. Fc-dependent phagocytosis correlated more strongly with neutralization than any other feature (Spearman’s rank correlation coefficient of 0.502).

### 3.3 MOMP-specific antibody responses were not associated with resistance to infection

**Figure 4:**
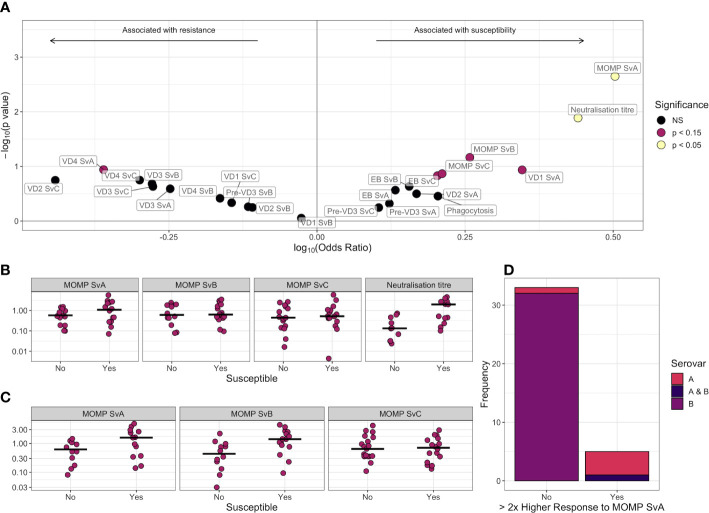
**(A)** Volcano plot for association between different antibody features and susceptibility to infection. Log-transformed odds ratio is shown on the *x* axis, with a log_10_(Odds ratio) > 0 indicating the association with susceptibility in a logistic regression model where age, sex, and ethnicity were included as covariates. On the *y* axis, features more significantly associated with susceptibility have a greater value of –log_10_(*p*-value). Points are colored by significance level: non-significant (NS) in black, *p* < 0.15 in pink, and *p* < 0.05 in yellow. **(B)** Comparison of scaled anti-MOMP SvA IgG, anti-MOMP SvB IgG, anti-MOMP SvC IgG, and neutralization titer between susceptible and resistant participants. The median is indicated by a horizontal line. Values are shown on a log_10_ transformed axis. **(C)** Comparison of scaled anti-MOMP SvA IgG, anti-MOMP SvB IgG, and anti-MOMP SvC IgG between susceptible and resistant participants for an ELISA carried out at a different site. The median is indicated by a horizontal line. Values are shown on a log_10_ transformed axis. **(D)** Serovars that participants with high baseline anti-MOMP SvA IgG (>2× response to MOMP SvB IgG) became infected with, versus serovars that participants without high baseline anti-MOMP SvA IgG became infected with.

A logistic regression model was used to assess the association of each feature with susceptibility to infection over the following 6 months, adjusting for age, sex, and ethnicity (**Figure 4A** and **Table 2**). No features were significantly associated with resistance (*p* < 0.05), but IgG responses towards MOMP serovar A (*p* = 0.0022, false discovery rate = 0.052) and neutralization titer (*p* = 0.013, false discovery rate = 0.15) were higher in susceptible individuals (**Figure 4B**). The higher levels of IgG against MOMP serovar A in susceptible individuals was validated in an ELISA carried out at a different site, and carried out at a single serum dilution rather than by area under the dilution curve (**Figure 4C**; *p* = 0.0003, logistic regression model).

**Table 2:** Association between different antibody features and susceptibility to infection.

Antibody feature	Log_10_(Odds ratio) (95% confidence intervals)	*p*-value	False discovery rate
Higher in susceptible individuals			
MOMP SvA	0.5 (0.23 to 0.89)	0.0022	0.052
Neutralization titer	0.44 (0.15 to 0.89)	0.013	0.15
MOMP SvB	0.26 (−0.00077 to 0.56)	0.068	0.45
VD1 SvA	0.35 (−0.022 to 0.91)	0.12	0.45
MOMP SvC	0.21 (−0.03 to 0.53)	0.14	0.45
EB SvC	0.2 (−0.036 to 0.52)	0.15	0.45
EB SvB	0.16 (−0.072 to 0.45)	0.23	0.45
EB SvA	0.13 (−0.09 to 0.39)	0.27	0.45
VD2 SvA	0.17 (−0.16 to 0.53)	0.32	0.49
Phagocytosis	0.2 (−0.21 to 0.76)	0.35	0.5
Pre-VD3 SvA	0.12 (−0.23 to 0.49)	0.48	0.58
Pre-VD3 SvC	0.1 (−0.26 to 0.49)	0.57	0.59
Higher in resistant individuals			
VD4 SvA	−0.36 (−0.91 to 0.043)	0.11	0.45
VD2 SvC	−0.44 (−1.2 to 0.068)	0.18	0.45
VD4 SvC	−0.3 (−0.81 to 0.094)	0.18	0.45
VD3 SvB	−0.28 (−0.8 to 0.12)	0.21	0.45
VD3 SvC	−0.28 (−0.83 to 0.12)	0.23	0.45
VD3 SvA	−0.25 (−0.77 to 0.14)	0.26	0.45
VD4 SvB	−0.16 (−0.55 to 0.21)	0.38	0.52
VD1 SvC	−0.14 (−0.57 to 0.23)	0.46	0.58
Pre-VD3 SvB	−0.12 (−0.51 to 0.27)	0.55	0.59
VD2 SvB	−0.11 (−0.5 to 0.26)	0.56	0.59
VD1 SvB	−0.026 (−0.41 to 0.33)	0.88	0.88

Log_10_(Odds ratio) > 0 indicating association with susceptibility in a logistic regression model where age, sex, and ethnicity were included as covariates. 95% confidence intervals are indicated.

For 38 individuals, both serovar and serology data were available. While IgG responses against MOMP serovar B were on average similar to MOMP serovar A (median 1.07 vs. 1.01), six of these individuals had a baseline response against serovar A over twice the magnitude of their response to B. These individuals were significantly more likely to later become infected with serovar A or both A and B (**Figure 4D**, *p* = 1 × 10^−5^, Fisher test), indicating that even with high levels of serum antibody, children can become re-infected with the same serovar. Bacterial load was not significantly different between the first, second, and third infection episodes (**Figure S3**), suggesting that previous infection has little impact on immunity within the time scale of the study.

### 3.4 Supervised machine learning is not able to distinguish between susceptible and resistant participants based on antibody profile

**Figure 5:**
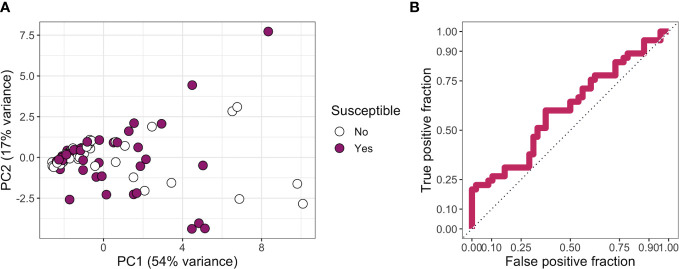
**(A)** Principal components 1 and 2 for a principal component analysis carried out on all features. Missing data were imputed using k-nearest neighbors. Points are colored by whether a participant was classed as resistant or susceptible. **(B)** True-positive rate against false-positive rate for a partial least squares model used to classify participants into “susceptible” or “resistant” categories.

Finally, as no individual antibody features were associated with resistance, we next investigated whether those who were resistant to infection had a broadly distinct antibody profile from those who were susceptible. Following imputation of missing data using k-nearest neighbors, principal component analysis based on all features did not reveal any major difference in overall serological profile between groups (**Figure 5A**). Tenfold cross-validation of a partial least squares model gave a specificity of 71%, a sensitivity of 36%, and an ROC of 0.58 in distinguishing susceptible from resistant individuals (**Figure 5B**).

Collectively, our results indicate that neither individual antibody features (*p* < 0.05) nor overall antibody profile (ROC of 0.58) is associated with protection against ocular *C. trachomatis* infection.

## 4 Discussion

We used a novel definition of susceptibility to identify whether antigen-specific or functional antibody responses were associated with susceptibility to ocular *C. trachomatis* infection. Interestingly, compared with male children, female children became more often infected when the infection burden in their family compound was lower. Although this has not previously been observed for infection, this is consistent with previous studies finding a greater burden of intense trachomatous inflammation, scarring, and trichiasis in female children, even at a young age (32–35). This is generally thought to be due to the caring responsibilities of women and female children, resulting in more time in proximity to the age group with the highest infection burden: children aged 5 and under (36).

Overall, the serological profiles of those who were susceptible and resistant were relatively similar, with a partial least squares model only performing slightly better than chance in classifying participants. Serum MOMP and EB IgG responses to different *C. trachomatis* serovars closely correlated to one another, although the SvB VD1 peptide was a notable outlier. This is likely due to SvB being the most antigenically distant of the three ocular serovars, with a deletion of two residues and differing C-terminus in the VD1 region (**Table S1**).

Serum MOMP and EB IgG responses were generally higher in those considered susceptible. This is consistent with previous studies finding that those with high levels of ocular *C. trachomatis-*specific IgG were at a higher risk of subsequently developing active trachoma (8) and that serum IgG titers to EBs are higher in participants with trachomatous scarring (9). Similarly for urogenital *C. trachomatis* infections, even after adjusting for confounding factors, anti-EB serum IgG is associated with a 3.63-fold higher risk of infection (37).

One potential explanation for the results observed here is that some unmeasured environmental or innate risk factor is increasing both past infections, which is reflected in high MOMP/EB IgG responses, and further infections after baseline. For example, poor facial cleanliness (38) and presence of flies (39) are risk factors for active trachoma, while genetic polymorphisms in TNF-α, IFNγ, IL-10, IL8, and CSF2 have been linked to risk of trachomatous scarring (40–44). It is unclear whether participants with poor innate immune responses or high exposure may have had more prolonged infections in the past, resulting in a greater antibody response.

However, we do not rule out the possibility of antibody-dependent enhancement, most famously associated with dengue fever. In secondary dengue virus infections, cross-reactive poorly neutralizing antibodies against a heterologous serotype can enhance infection of Fc-receptor-expressing cells (45). Like dengue, *C. trachomatis* is an intracellular pathogen with multiple circulating serovars. Here, we found that antibody-dependent phagocytosis was somewhat higher in susceptible individuals, albeit only in the small subgroup for which this assay was performed. Furthermore, serum IgG from women with a recent urogenital *C. trachomatis* infection, or monoclonal antibodies against *C. trachomatis*, can, in some cases, enhance *in vitro* uptake of EBs, presumably *via* an Fc receptor on target cells (46). On the other hand, we found neutralizing antibodies to also be higher in susceptible individuals, and there was no evidence of increased *C. trachomatis* burden in secondary infections.

It is clear that infection-induced anti-MOMP and EB IgG, at least at the levels reached by the children in our cohort, does not protect against re-infection. One possibility is that as the children in our study were relatively young, their IgG may not have yet reached a protective threshold. Another possibility is that the scope of the assays, in focusing on EB and MOMP-specific IgG responses in serum, was too limited. For example, it has previously been found that serum anti-EB IgA titers are higher in healthy controls compared to those with trachomatous scarring (9), and anti-*C. trachomatis* ocular IgA titers are (non-significantly) higher in those protected from acquiring trachomatous disease (8). Antibody responses to less well characterized antigens could also be protective. For example, protein arrays have identified anti-CT442 antibody responses as potentially protective, as they are higher in those without scarring in a trachoma-endemic area (31). Furthermore, local ocular responses may have a different effect than serum responses. Finally, *C. trachomatis*-specific CD4^+^ IFN-γ responses have been shown to be highly important for protection against re-infection, and therefore, humoral responses may play a redundant role in immunocompetent individuals (47).

This study was limited in that certain assays were not carried out for every participant, and systemic rather than ocular responses were measured. However, as participants were longitudinally monitored for infection following baseline blood sample collection, we were uniquely placed to examine how antibody profile preceding infection relates to susceptibility. We find that infection-induced antibody responses towards MOMP do not appear to be protective in this setting, suggesting that natural immunity may be mediated by other mechanisms, or develop at an older age than the participants included in this study.

## Data availability statement

The original contributions presented in the study are included in the article/**Supplementary Material**. Further inquiries can be directed to the corresponding author.

## Ethics statement

The studies involving human participants were reviewed and approved by Gambian Government-Medical Research Council Ethics Committee and the London School of Hygiene and Tropical Medicine Ethics Committee. Written informed consent to participate in this study was provided by the participants’ legal guardian/next of kin.

## Author contributions

AB led the statistical and multivariate analysis, with significant contribution from MH, IR, and JD. AB wrote the first draft, which was reviewed and edited by IR, HP, NF, AH, HJ, PM, MR, AO, RB, DM, FF, JD, and MH. NF, HJ, PM, RB, and DM were involved in clinical study sample collection. IR, JD, and AO designed the experiments and methodology, and IR and AO provided reagents. Experiments were carried out by IR (peptide and MOMP ELISA, phagocytosis, and neutralization assays) HP (EB and MOMP ELISA), AH, and MR (*C. trachomatis* sequencing). All authors contributed to the article and approved the submitted version.
