# Supplementary material for: A systems serology approach to the investigation of infection-induced antibody responses and protection in trachoma
Source: Front Immunol. 2023 May 23;14:1178741. doi: 10.3389/fimmu.2023.1178741 (PMC10242090; doi:10.3389/fimmu.2023.1178741)

## Supplementary figures

Table S1: MOMP peptides used for IgG ELISA with serum samples

| Name | Residues | Sequence |
| --- | --- | --- |
| VD1 SvA | 83-104 | QMGAAPTTSDVAGLEKDPVTNV |
| VD1 SvB | 83-102 | QMGAKPTATTGNATAPSTLT |
| VD1 SvC | 83-104 | QMGAAPTTSDVAGLQNDPTTNV |
| VD2 SvA | 165-185 | TQSSGFDTANLVPNTALNQAV |
| VD2 SvB | 163-183 | ENQTKVSNGTFVPNMSLDQSV |
| VD2 SvC | 165-185 | TQSSSFNTAKLIPNTALNEAV |
| PreVD3 SvA | 236-255 | EFTINKPKGYVGAEFPLDIT |
| PreVD3 SvB | 234-253 | EFTINKPKGYVGKELPLDLT |
| PreVD3 SvC | 236-255 | EFTINKPKGYVGAEFPLNIT |
| VD3 SvA | 246-265 | VGAEFPLDITAGTEAATGTK |
| VD3 SvB | 244-263 | VGKELPLDLTAGTDAATGTK |
| VD3 SvC | 246-265 | VGAEFPLNITAGTEAATGTK |
| VD4 SvA | 316-335 | VLDTTTLNPTIAGKGTVVSS |
| VD4 SvB | 314-333 | IFDVTTLNPTIAGAGDVKTS |
| VD4 SvC | 316-335 | ILDVTTLNPTIAGKGSVVSA |

Figure S1: Location of the 19 compounds, within five villages in The Gambia, from which serum samples were collected. Each compound is represented by a point, with the village indicated by color and label.


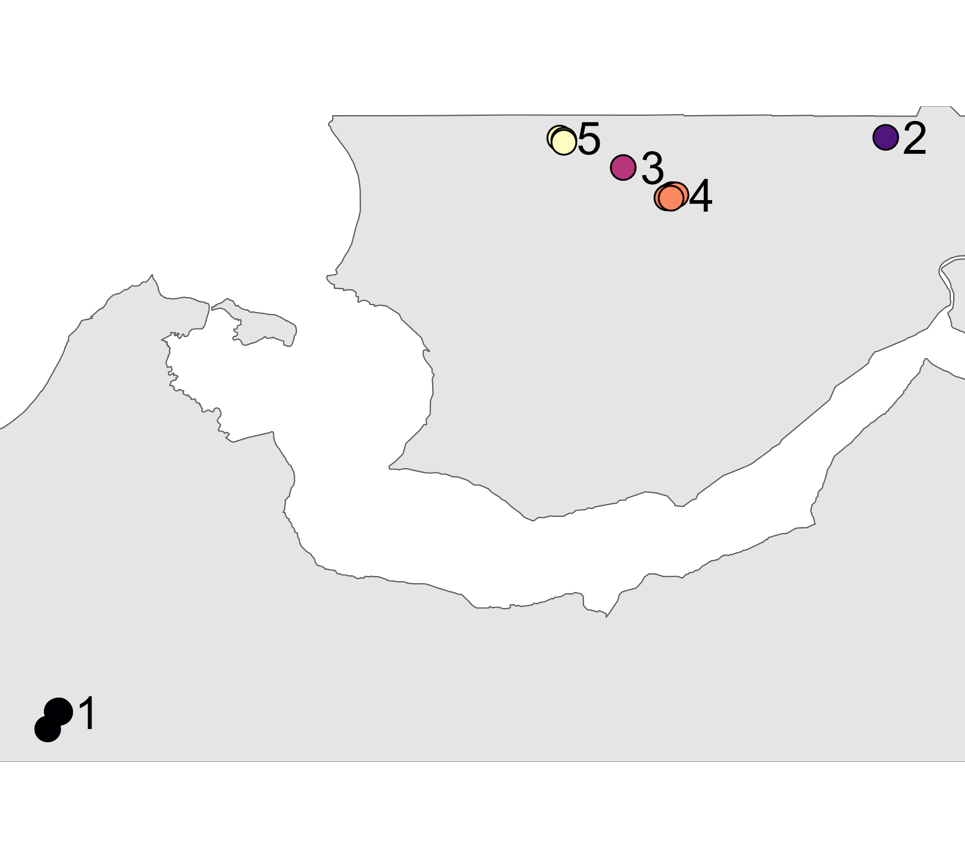


Figure S2: Illustration of how, for each participant, susceptibility to infection was estimated. For each participant, we estimated what infection burden their compound would need to reach before they themselves became infected. For example 1, they were infected at every time point, and therefore their infection threshold was set to 0. For example 6, they were not infected at any time points, and therefore their infection threshold was set to 100. Example participant 3 was not infected when ≤ 50% of sampled participants in their compound were infected, but did become infected when ≥ 75% sampled participants in their compound were infected. Therefore their infection threshold was set at 63%


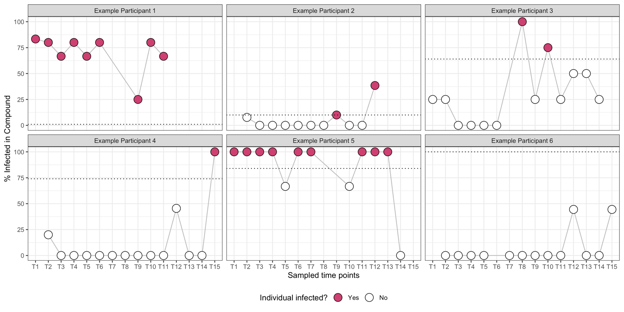


Table S2: Characteristics of “susceptible” and “resistant” participants for the 93 samples on which serological assays were performed, subset by village.

| Village | Aspect | Susceptible | Resistant |
| --- | --- | --- | --- |
| 1 | N | 23 | 7 |
|  | Age | 7 (IQR 5-10.5) | 9 (IQR 7.5-11.5) |
|  | % Female | 60.9 | 14.3 |
|  | Ethnicity | Manjago 100 % | Manjago 100 % |
| 2 | N | 3 | 17 |
|  | Age | 13 (IQR 9.5-13.5) | 11 (IQR 7-13) |
|  | % Female | 0 | 23.5 |
|  | Ethnicity | Mandinka 67 % Wolof 33 % | Mandinka 100 % |
| 3 | N | 3 | 14 |
|  | Age | 8 (IQR 7.5-8) | 6 (IQR 6-8) |
|  | % Female | 33.3 | 50 |
|  | Ethnicity | Serahule 100 % | Serahule 100 % |
| 4 | N | 9 | 7 |
|  | Age | 7 (IQR 5-12) | 7 (IQR 6-9) |
|  | % Female | 66.7 | 28.6 |
|  | Ethnicity | Mandinka 67 % Serer 11 % Wolof 22 % | Mandinka 100 % |
| 5 | N | 7 | 3 |
|  | Age | 6 (IQR 5.5-7.5) | 12 (IQR 10-13) |
|  | % Female | 85.7 | 33.3 |
|  | Ethnicity | Mandinka 86 % Wolof 14 % | Mandinka 100 % |

Figure S3: *C. trachomatis* 16S rRNA copies in the first, second and third infection episodes. Each point represents one sample, with grey lines joining samples from the same participant. P values for a paired-wilcoxon test are indicated.


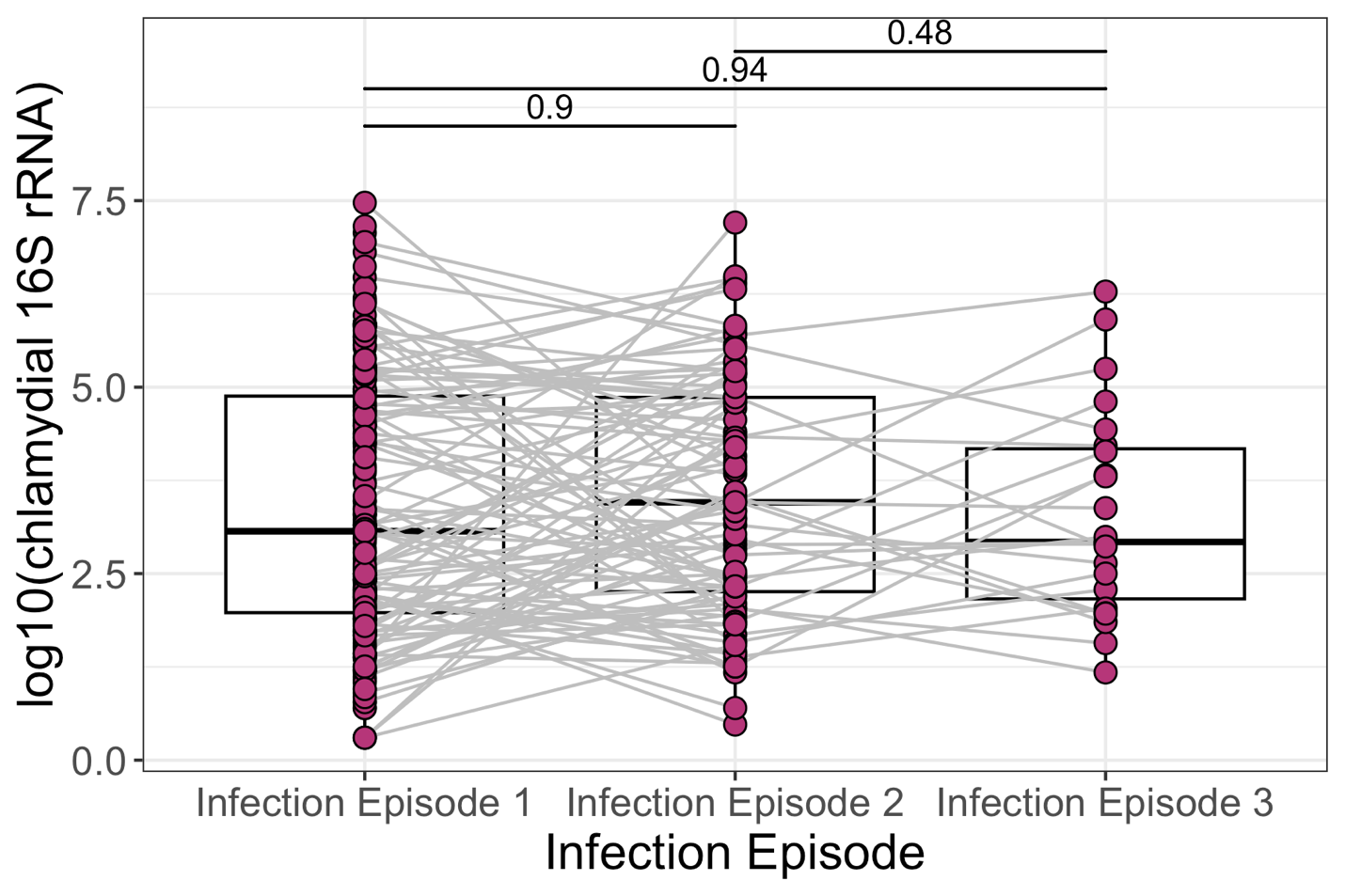

Supplement: Supplementary file 3 [file DataSheet_3.docx]
